# Andrographolide Protects against Aortic Banding-Induced Experimental Cardiac Hypertrophy by Inhibiting MAPKs Signaling

**DOI:** 10.3389/fphar.2017.00808

**Published:** 2017-11-14

**Authors:** Qing Q. Wu, Jian Ni, Ning Zhang, Hai H. Liao, Qi Z. Tang, Wei Deng

**Affiliations:** ^1^Department of Cardiology, Renmin Hospital of Wuhan University, Wuhan, China; ^2^Cardiovascular Research Institute, Wuhan University, Wuhan, China; ^3^Hubei Key Laboratory of Cardiology, Wuhan, China; ^4^Department of Cardiology, The Fifth Affiliated Hospital of Xinjiang Medical University, Ürümqi, China

**Keywords:** andrographolide, cardiac hypertrophy, cardiomyocyte, fibroblast, MAPKs

## Abstract

Despite therapeutic advances, heart failure-related mortality rates remain high. Therefore, understanding the pathophysiological mechanisms involved in the remodeling process is crucial for the development of new therapeutic strategies. Andrographolide (Andr), a botanical compound, has potent cardio-protective effects due to its ability to inhibit mitogen-activated protein kinases (MAPKs). Andr has also been shown to inhibit inflammation and apoptosis, which are factors related to cardiac hypertrophy. Our aim was to evaluate the effects of Andr on cardiac hypertrophy and MAPKs activation. Thus, mice were subjected to aortic banding (AB) with/without Andr administration (25 mg/kg/day, orally). Cardiac function was accessed by echocardiography and hemodynamic parameters. Our results showed that Andr administration for 7 weeks decreased cardiac dysfunction and attenuated cardiac hypertrophy and fibrosis in AB mice. Andr treatment induced a strong reduction in the transcription of both hypertrophy (ANP, BNP, and β-MHC) and fibrosis related genes (collagen I, collagen III, CTGF, and TGFβ). In addition, cardiomyocytes treated with Andr showed a reduced hypertrophic response to angiotensin II. Andr significantly inhibited MAPKs activation in both mouse hearts and cardiomyocytes. Treatment with a combination of MAPKs activators abolished the protective effects of Andr in cardiomyocytes. Furthermore, we found that Andr also inhibited the activation of cardiac fibroblasts via the MAPKs pathway, which was confirmed by the application of MAPKs inhibitors. In conclusion, Andr was found to confer a protective effect against experimental cardiac hypertrophy in mice, suggesting its potential as a novel therapeutic drug for pathological cardiac hypertrophy.

## Introduction

Heart failure (HF) is a burgeoning problem that affects more than 20 million individuals worldwide (Tham et al., 2015). Cardiac hypertrophy is a response of the heart to increased workload (such as aortic stenosis, hypertension, and dilated cardiomyopathy) and various insults (such as myocarditis and myocardial infarction). It typically progresses to heart failure (Lyon et al., 2015; Shimizu and Minamino, 2016). Increased cardiomyocyte size and thickening of ventricular walls are the main features of cardiac hypertrophy (Lyon et al., 2015; Shimizu and Minamino, 2016). Maladaptive hypertrophy involves increased cardiomyocyte hypertrophy and, apoptosis as well as increased fibroblast activation, which are associated with reduced systolic function and increased heart stiffness (Samak et al., 2016; Shimizu and Minamino, 2016). Left ventricular hypertrophy is positively correlated with an increased risk of adverse cardiovascular events (Hill and Olson, 2008). Accumulating evidence indicates that multiple signaling pathways participate in the progression of cardiac hypertrophy, such as insulin growth factor/PI3K/Akt, protein kinase C, and mitogen-activated protein kinases (MAPKs), β-adrenergic receptor, calcineurin/NFAT, and Ca^2+^/CaMKII signaling (Hou and Kang, 2012; Tham et al., 2015). However, the complexity of the mechanism underlying the transition from hypertrophic processes to heart failure and the difficulty in reversing cardiac hypertrophy contribute to the high mortality rates of heart failure. Therefore, new pharmacological agents that selectively inhibit the progression of cardiac hypertrophy are of great therapeutic interest.

MAPKs are involved in diverse biological events, including proliferation, differentiation, metabolism, motility, survival, and apoptosis (Liu and Molkentin, 2016). MAPKs subfamilies include ERK1/2, c-Jun NH2-terminal kinases (JNK), and P38 kinase (Liu and Molkentin, 2016). ERK1/2 signaling co-ordinates the eccentric and concentric growth of the heart. In addition, ERK autophosphorylation at Thr188 facilitates ERK1/2 activity toward nuclear targets, which is a critical event in the induction of ERK-mediated cardiac hypertrophy in response to various stimuli (Lorenz et al., 2009; Li W.M. et al., 2017). JNK can shuttle between the cytoplasm and the nucleus to exert its effects. Overactivation of JNK leads to a hypertrophic phenotype, and abrogation of JNK activity attenuates endothelin-1 (ET-1) and the pressure overload-induced hypertrophic response (Li W.M. et al., 2017). P38 is rapidly activated within a few minutes of exposure to aortic pressure or volume overload. Cardiac-specific overexpression of P38 leads to enhanced cardiac hypertrophy in response to pressure overload (Liao et al., 2001). The complexity of the signaling transduction network makes it impossible and imprudent to label any molecule as definitively ‘bad’ or ‘good.’ Thus, by focusing on network interactions rather than individual signaling molecules, we have a better chance of influencing the outcome.

*Andrographis paniculata* is a traditional medicinal herb that is used in China (Banerjee et al., 2017). Andrographolide is the major bioactive component of *Andrographis paniculata*. To date, studies have reported many pharmacological effects of Andr, such as anti-inflammatory (Ren et al., 2016), anti-oxidant (Chen et al., 2014), antihyperglycemic (Li et al., 2015), and hepatoprotective properties (Pan et al., 2017). Recent studies have found that Andr inhibits aconitine-induced arrhythmia by inhibiting voltage-gated Na^+^ (I_Na_), and Ca^2+^ (I_CaL_) (Zeng et al., 2017). By inhibiting IκB phosphorylation and NF-κB activation, Andr relieves lipopolysaccharide-induced cardiac malfunctions in mice (Zhang et al., 2015). Andr has also been reported to protect against hypoxia/reoxygenation injury in cardiomyocytes by regulating glutathione levels (Woo et al., 2008). Recently, Hsieh YL reported that *Andrographis paniculata* extract attenuates pathological cardiac hypertrophy and apoptosis in high-fat diet-fed mice (Hsieh et al., 2016). All these findings suggest the cardiac protective effects of Andr. Andr was also reported to inhibit MAPKs in many disease models, including acute lung injury (Peng et al., 2016), rheumatoid arthritis (Li Z.Z. et al., 2017), Alzheimer’s disease (Yang et al., 2017), and ischemic stroke models (Yen et al., 2016). These studies indicate that Andr may exert anti-hypertrophic effects by regulating MAPKs. Aortic banding (AB) is a reliable model of left ventricular pressure overload for the study of the progression from compensated hypertrophy to heart failure, thus enabling the monitoring of cardiac remodeling (Martin et al., 2012). It is known that pressure overload can activate the renin-angiotensin system and induce the release of angiotensin II (Ang II), which activates the Gα (q) protein-coupled receptor signaling pathway (Wu et al., 2015). Thus, Ang II was used *in vitro* to induce cardiac hypertrophy in cardiomyocytes. The aim of our study was to explore the effects of Andr on pressure overload-induced cardiac hypertrophy, and fibrosis as well as the underlying mechanisms.

## Materials and Methods

### Chemicals

Andrographolide was purchased from Shanghai Winberb Medical S&T Development Co. Ltd. (Shanghai, China) with a purity >98% as determinated by high-performance liquid chromatography analysis.

### Animals

Eight to ten weeks old male C57/BL6 mice were purchased from the Institute of Laboratory Animal Science, CAMS&PUMC (Beijing, China). Mice were housed in the Cardiovascular Research Institute of Wuhan University (Wuhan, China) with controlled temperature and humidity. AB surgery was performed as previously described (Wu et al., 2015, 2016). After 1 week of AB or sham surgery, the animals were treated with Andr daily (25 mg/kg body weight/day, oral gavage, suspended in 0.5% carboxymethyl cellulose solution) until 8 weeks after surgery. Four groups were included: the vehicle-sham group (veh-sham, *n* = 15), the Andr-sham group (*n* = 15), the vehicle-AB group (veh-AB, *n* = 15), and the Andr-AB group (*n* = 15). All the experimental procedures were in accordance with the institutional guidelines and approved by the Animal Care and Use Committee of Renmin Hospital of Wuhan University (Approval number: WHRM 2015 W01; exact date of approval: January 1st, 2015).

### Echocardiography

Cardiac functions were measured in our laboratory (Wu et al., 2015, 2016). Briefly, echocardiography was performed on anesthetized (1.5% isoflurane) mice using a MyLab 30CV ultrasound system (Biosound Esaote, Genoa, Italy) with a 10-MHz linear array ultrasound transducer.

Parasternal short axis images were obtained at the level of the mid-papillary muscle in M-mode. Left ventricular (LV) dimensions from five consecutive cardiac cycles were measured and averaged, including LVEDs, LVEDd, end-diastolic LVPWd, and end-systolic LVPW (LVPWs). Fractional shortening (FS) and LV ejection fraction (EF) were calculated using the LVEDs and LVEDd values.

### Measurement of Hemodynamic Parameters

Hemodynamic parameters were measured in our laboratory (Wu et al., 2015, 2016). Briefly, hemodynamics were measured in anesthetized (1.5% isoflurane) mice using cardiac catheterization. A microtip catheter transducer (SPR-839; Millar Instruments, Houston, TX, United States) was inserted into the right carotid artery and advanced into the LV. Data including HR, end-diastolic pressure (EDP), end-systolic pressure (ESP), dP/dt max, and dP/dt min were analyzed.

### Histological Analysis

Heart slides were obtained using previously described methods (Wu et al., 2015, 2016). HE staining was performed to assess the cardiomyocyte cross-sectional area (CSA) and to observe the morphology of striated muscle. Sirius red in saturated picric acid (PSR) staining was used to determine interstitial fibrosis. A quantitative digital image analysis system (Image-Pro Plus, version 6.0; Media Cybernetics, Rockville, MD, United States) was used to trace a single myocyte (100–200 myocytes in each group).

### Quantitative Real-Time Polymerase Chain Reaction (RT-PCR)

Total RNA and cDNA were prepared previously described (Wu et al., 2015, 2016).

We performed 20-μl reactions according to the manufacturer’s protocol with the following cycling parameters: 95°C for 5 min; 45 cycles of 95°C for 10 s, 60°C for 10 s, and 72°C for 10 s; 95°C for 5 s; 60°C for 1 min; 97°C for 0.11 s; and 40°C for 10 min. The results were analyzed with the 2^-ΔΔCt^ method and normalized to GAPDH gene expression. The primer sequences used in the RT-PCR experiment are listed in **Table 1**.

**Table 1:** Primer sequences for RT-PCR.

mRNA	Forward	Reverse
ANP^a^	ACCTGCTAGACCACCTGGAG	CCTTGGCTGTTATCTTCGGTACCGG
BNP	GAGGTCACTCCTATCCTCTGG	GCCATTTCCTCCGACTTTTCTC
β-MHC^a^	CCGAGTCCCAGGTCAACAA	CTTCACGGGCACCCTTGGA
Collagen I^a^	AGGCTTCAGTGGTTTGGATG	CACCAACAGCACCATCGTTA
Collagen III^a^	AAGGCTGCAAGATGGATGCT	GTGCTTACGTGGGACAGTCA
CTGF^a^	AGGGCCTCTTCTGCGATTTC	CTTTGGAAGGACTCACCGCT
TGFβ^a^	ATCCTGTCCAAACTAAGGCTCG	ACCTCTTTAGCATAGTAGTCCGC
GAPDH^a^	ACTCCACTCACGGCAAATTC	TCTCCATGGTGGTGAAGACA
ANP^b^	AAAGCAAACTGAGGGCTCTGCTCG	TTCGGTACCGGAAGCTGTTG CA
BNP^b^	CAGCAGCTTCTGCATCGTGGAT	TTCCTTAATCTGTCGCCGCTGG
β-MHC^b^	TCTGGACAGCTCCCCATTCT	CAAGGCTAACCTGGAGAAGATG
Collagen I^b^	GAGAGAGCATGACCGATGGATT	TGGACATTAGGCGCAGGAA
Collagen III^b^	AAGGGCAGGGAACAACTGAT	GTGAAGCAGGGTGAGAAGAAAC
CTGF^b^	GGAAGACACATTTGGCCCTG	GCAATTTTAGGCGTCCGGAT
GAPDH^b^	GACATGCCGCCTGGAGAAAC	AGCCCAGGATGCCCTTTAGT

*Sequences are listed 5′–3′. ^a^ Primers used in mouse experiments. ^b^ Primers used for H9c2 rat cardiomyocytes and neonatal rat cardiac fibroblasts.*

### Western Blotting

Total protein was extracted from heart tissue and cells. The Bicinchoninic Acid Protein Assay kit (Thermo Fisher Scientific, Inc., Waltham, MA, United States) was used to measure the concentration of protein. The protein lysates were separated by 10% SDS-PAGE and transferred to PVDF membranes as previously described (Wu et al., 2015, 2016). The next day, blots were incubated with secondary antibodies for 1 h. A two-color infrared imaging system (Odyssey; LI-COR Biosciences, Lincoln, NE, United States) was used to scan the protein blots. The primary antibodies used in this study were as follows: phospho-ERK1/2 Thr202/Tyr204 [1:1000, #4370, Cell Signaling Technology (CST), Danvers, MA, United States], ERK1/2 (1:1000, #4695, CST), phospho-P38Thr180/Tyr182 (1:1000, #4511, CST), P38 (1:1000, #9212, CST), phosphor-JNK1/2Thr183/Tyr185 (1:1000, #4668, CST), JNK1/2 (1:1000, #9285, CST), smad4 (1:1000, #9515, CST), and GAPDH (1:1000, #2118, CST).

### Cell Culture

H9c2 cells were prepared according to our laboratory’s protocols (Wu et al., 2015). The H9c2 cardiomyocytes were obtained from the Cell Bank of the Chinese Academy of Sciences, in Shanghai, China and cultured in DMEM (C11995; GIBCO, Thermo Fisher, Waltham, MA, United States) supplemented with 10% fetal bovine serum (10099; GIBCO) in an atmosphere containing 5% CO_2_ inside a humidified incubator (SANYO 18 M, Osaka, Japan) at 37°C. The cells were divided into four groups: the control group (CON), the Ang II (1 μM, Sigma) treatment group, the Andr (12.5, 25, or 50 μM) group, and the Ang+Andr (12.5, 25, or 50 μM) group. After treatment for 24 h, cells from six wells were harvested for PCR analysis, while cells from 24 wells were used for immunofluorescence staining.

### Isolation and Culture of Cardiac Fibroblasts

Neonatal rat cardiac fibroblasts were prepared to our laboratory’s protocols (Wu et al., 2017a). Briefly, neonatal rats that were born within 3 days were sacrificed, and the hearts were collected. The hearts were cut into 1-mm^3^ tissue and digested in 0.125% trypsin for 15 min at 34°C for a total of five times. The digestive fluid was collected and centrifuged. Cells were resuspended, filtered and then seeded onto 100-mm plates for 90 min. After removing the cardiomyocytes, the cardiac fibroblasts were cultured in DMEM/F12 containing 10% FBS at 37°C in a humidified incubator with 5% CO2. Before treatment with Ang II (1 μM) and Andr (12.5, 25, or 50 μM), the cells were cultured in 1% FBS for 12 h. After treatment for 24 h cells from six wells were harvested for PCR analysis while cells from 24 wells were used for immunofluorescence staining.

### Cell Counting Kit-8 Assay

Cell viability was evaluated using the cell counting kit (CCK)-8 assay, according to the manufacturer’s instructions. Briefly, 10 μl of CCK-8 solution was added to each well of a 96-well plate, and the absorbance was measured at 450 nm using an ELISA reader (Synergy HT, Bio-tek, Winooski, VT, United States) after a 4-h incubation. The effect of Andr on cell viability was expressed as the percentage of viable cells compared with that in the vehicle group, which was set at 100%.

### Immunofluorescence

Culture medium was discarded, and the cells were washed with PBS; then, 4% paraformaldehyde was used to fix the cells for 15 min at room temperature. After rinsing with PBS, the cells were permeabilized with 0.5% Triton X-100 and then rinsed with PBS three times for 5 min each. Primary antibodies against α-actinin (Millipore, 2207266, Darmstadt, Germany) and α-SMA (Abcam, ab7817) at a 1:100 dilution were added to H9c2 cells and cardiac fibroblasts, respectively, in a 24-well plate at 4°C overnight. The next day, sections were washed with PBS and then incubated with Alexa Fluor^®^ 488 goat anti-mouse IgG (H+L) antibodies for 60 min at 37°C. Finally, after washing with PBS, Slow Fade Gold antifade reagent with DAPI was used to seal the sections before observation and imaging with a fluorescence microscope. Image-Pro Plus 6.0 was used to analyze the images (*n* = 5 samples per group and *n* = 100+ cells were analyzed per group).

### Statistical Analysis

All values were presented as the mean ± SEM. SPSS 19.0 for Windows was used for the analysis. One-way ANOVA followed by *post hoc* Tukey test was performed for the data analysis. Two-way ANOVA followed by *post hoc* Tukey test was performed to evaluate the time-dependent anti-hypertrophic effects of Andr *in vitro*. A *p*-value < 0.05 (two-tailed) was considered statistically significant.

## Results

### Andr Improves Cardiac Function after Chronic Pressure Overload in Mice

**FIGURE 1:**
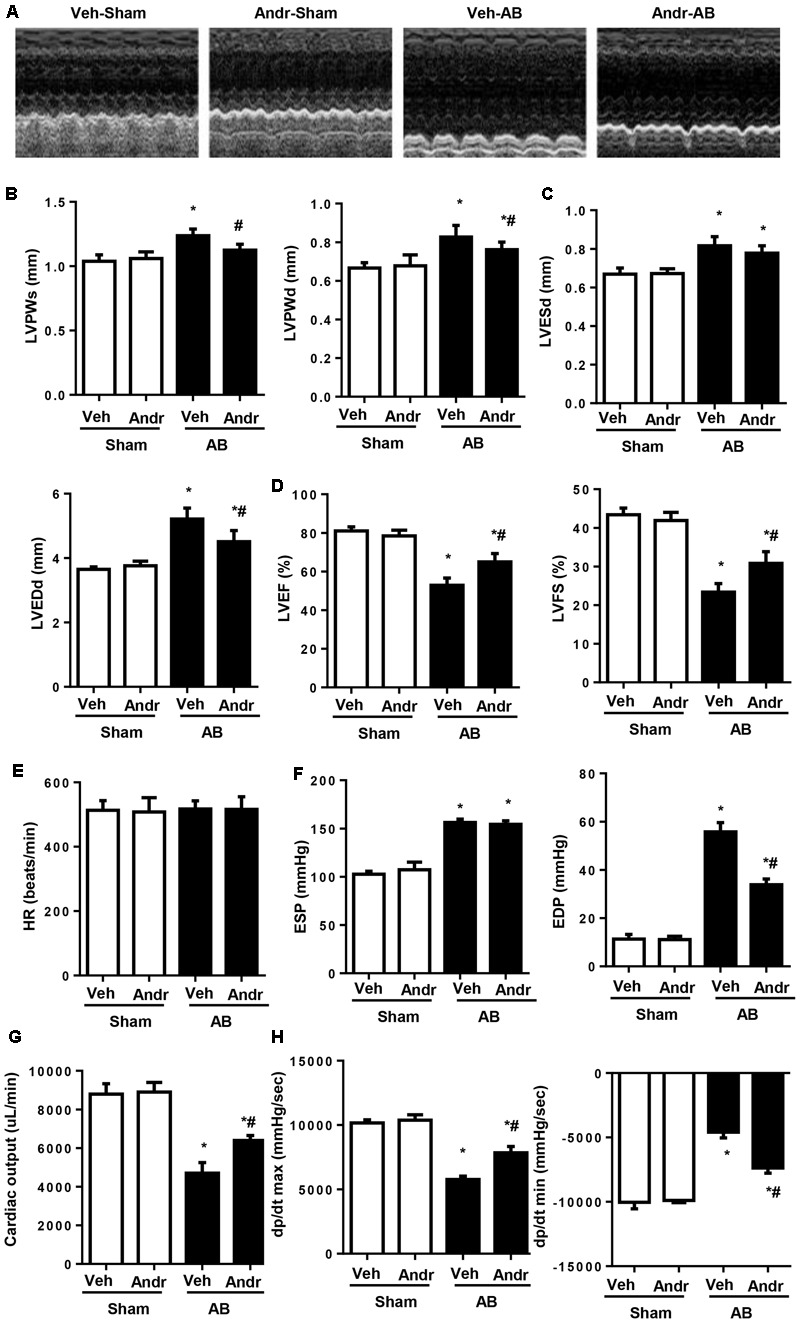
Andr improves cardiac function after chronic pressure overload in mice. **(A–D)** Echocardiography was performed at the end of the study (8 weeks) (*n* = 8). **(A)** Representative images. Andr attenuated the AB-induced increase in wall thickness [**B**, left ventricular (LV) posterior wall thickness at the end of systole (LVPWs) and, LV posterior wall thickness at the end of diastole (LVPWd)], and LV diameter **(C)**, including LVESd and LVEDd as well as attenuated AB-induced changes in ejection fraction (EF) and fractional shortening (FS) **(D)**. **(E–H)**. Hemodynamics analysis was performed at the end of the study (8 weeks) (*n* = 8). HR, heart rate; ESP, end-systolic pressure; EDP, end-diastolic pressure; CO, cardiac output; dP/dt max, maximal rate of pressure development; dP/dt min, minimal rate of pressure decay. ^∗^*P* < 0.05 compared with the corresponding sham group. ^#^*P* < 0.05 vs. the veh-AB group. AB, aortic banding.

To evaluate the effect of Andr on cardiac function, echocardiography was performed 8 weeks after AB (**Figure 1A**). The results showed that wall thickness (LVPWs, LVPWd, **Figure 1B**), chamber dilation (LVEDd, **Figure 1C**), and cardiac function (increased LVEF and LVFS, **Figure 1D**) were in the Andr-AB group than in the veh-AB group. There was no significant difference in HR among groups (**Figure 1E**). In addition, hemodynamics showed that Andr treatment improved systolic function and diastolic function after AB surgery (**Figures 1F–H**). No obvious differences were observed between the sham-vehicle and Andr mice.

### Andr Attenuates Cardiac Hypertrophy after Chronic Pressure Overload in Mice

**FIGURE 2:**
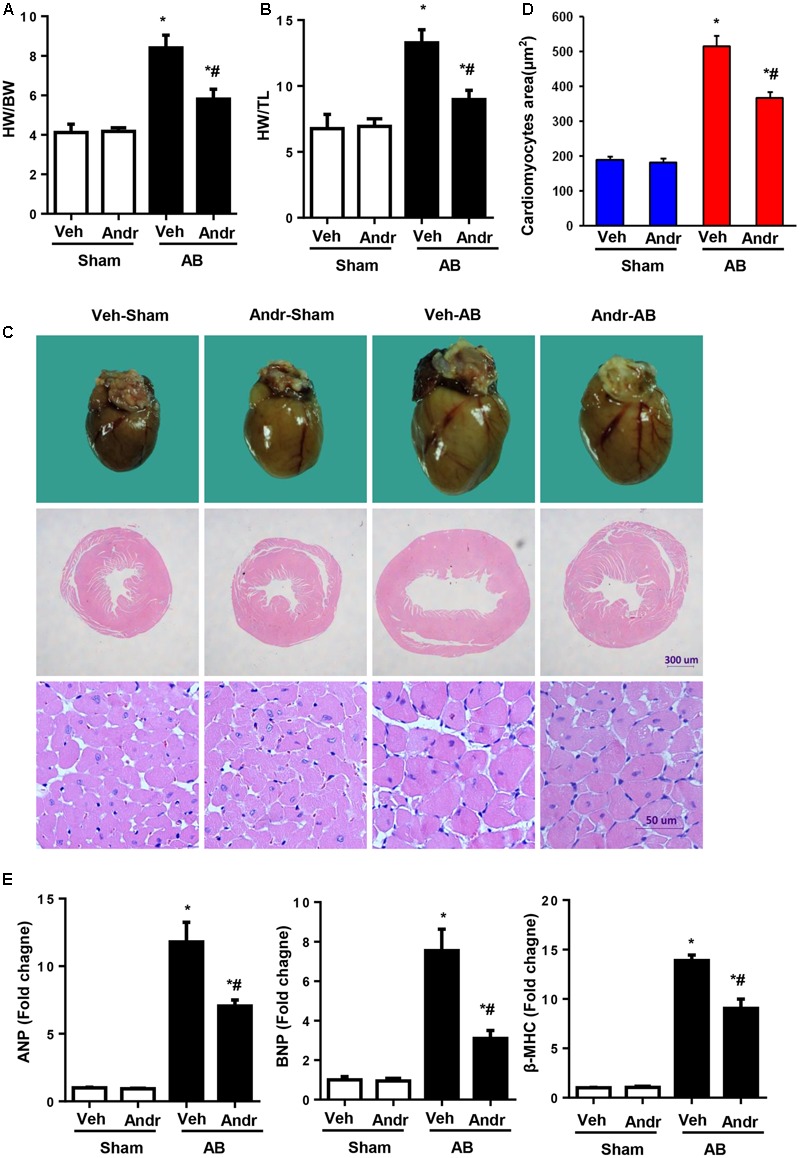
Andr attenuates cardiac hypertrophy after chronic pressure overload in mice. **(A,B)** Statistical results of the heart weight/body weight (HW/BW) ratio, and heart weight/tibial length (HW/TL) ratio (*n* = 8). **(C)** Gross hearts, and HE staining of hearts from sham and aortic banding (AB) mice at 8 weeks post-surgery. **(D)** Myocyte cross-sectional areas (CSAs) of the indicated groups (*n* = 200+). **(E)** Expression of transcripts for ANP, BNP, and β-myosin heavy polypeptide (MHC) induced by AB was determined by reverse transcription-polymerase chain reaction analysis (*n* = 6). The results are presented as a fold change, and the results are normalized to GAPDH gene expression. ^∗^*P* < 0.05 as compared with the corresponding sham group. ^#^*P* < 0.05 vs. the veh-AB group. AB, aortic banding.

Eight weeks after AB, Andr-treated mice presented decreased heart weight, which was accessed by heart weight to body weight ratios and heart weight to tibia length ratios (**Figures 2A,B**). Andr-treated mice also exhibited a strikingly decreased heart size and decreased cardiomyocyte CSA compared to vehicle treated animals (**Figures 2C,D**). Consistently, the expression of hypertrophic genes was significantly decreased in Andr-treated mice after AB (**Figure 2E**). Thus, Andr remarkably protected the heart from pressure overload-induced cardiac hypertrophy and dysfunction.

### Andr Attenuated Cardiac Fibrosis after Chronic Pressure Overload in Mice

**FIGURE 3:**
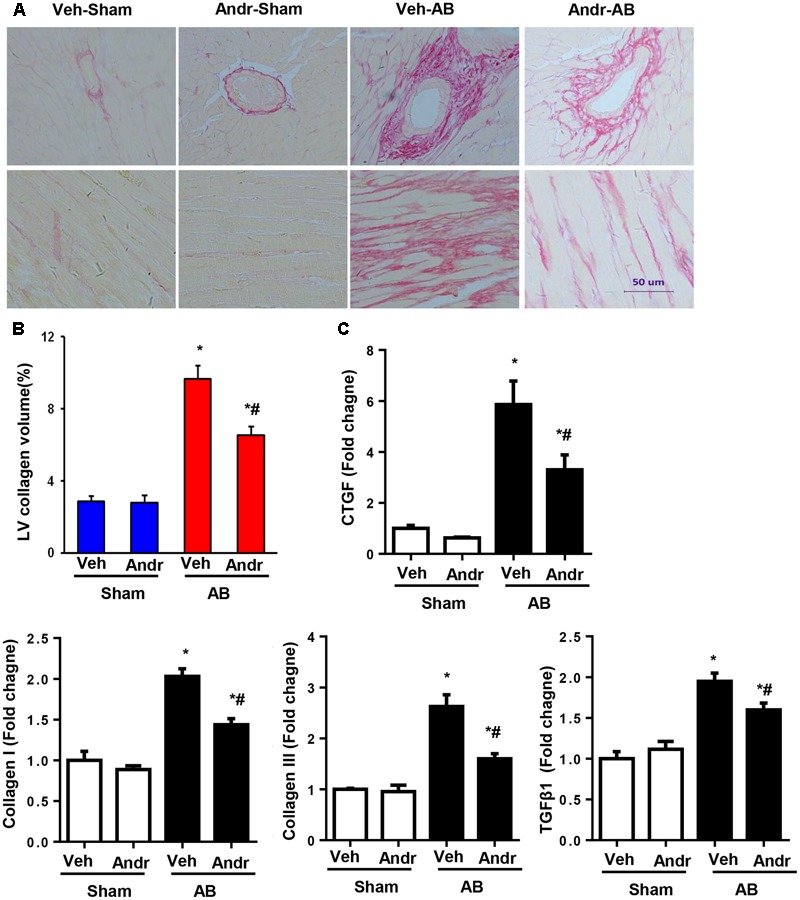
Andr attenuated cardiac fibrosis after chronic pressure overload in mice. **(A)** Left ventricular histological sections from the indicated groups were stained with PSR (*n* = 6). **(B)** Fibrotic areas in the histological sections were quantified using an image-analysis system. **(C)** The mRNA expression of CTGF, collagen I, collagen III, fibronectin, and TGFβ1 in the myocardium was analyzed in the indicated groups using reverse transcription-polymerase chain reaction (*n* = 6). The results are presented as a fold change, and the results are normalized to GAPDH gene expression. ^∗^*P* < 0.05 compared with the corresponding sham group. ^#^*P* < 0.05 vs. the veh-AB group.

Cardiac fibrosis, one of the main features of cardiac hypertrophy, was evaluated by PSR staining. After 8 weeks of AB, dramatic interstitial fibrosis was observed in all AB mice, but the extent of fibrosis was decreased in the hearts from the Andr-AB group (**Figures 3A,B**). The expression of myocardial pro-fibrotic genes was also down-regulated by Andr treatment (**Figure 3C**).

### Andr Suppresses Ang II Induced Cardiomyocyte Hypertrophy

**FIGURE 4:**
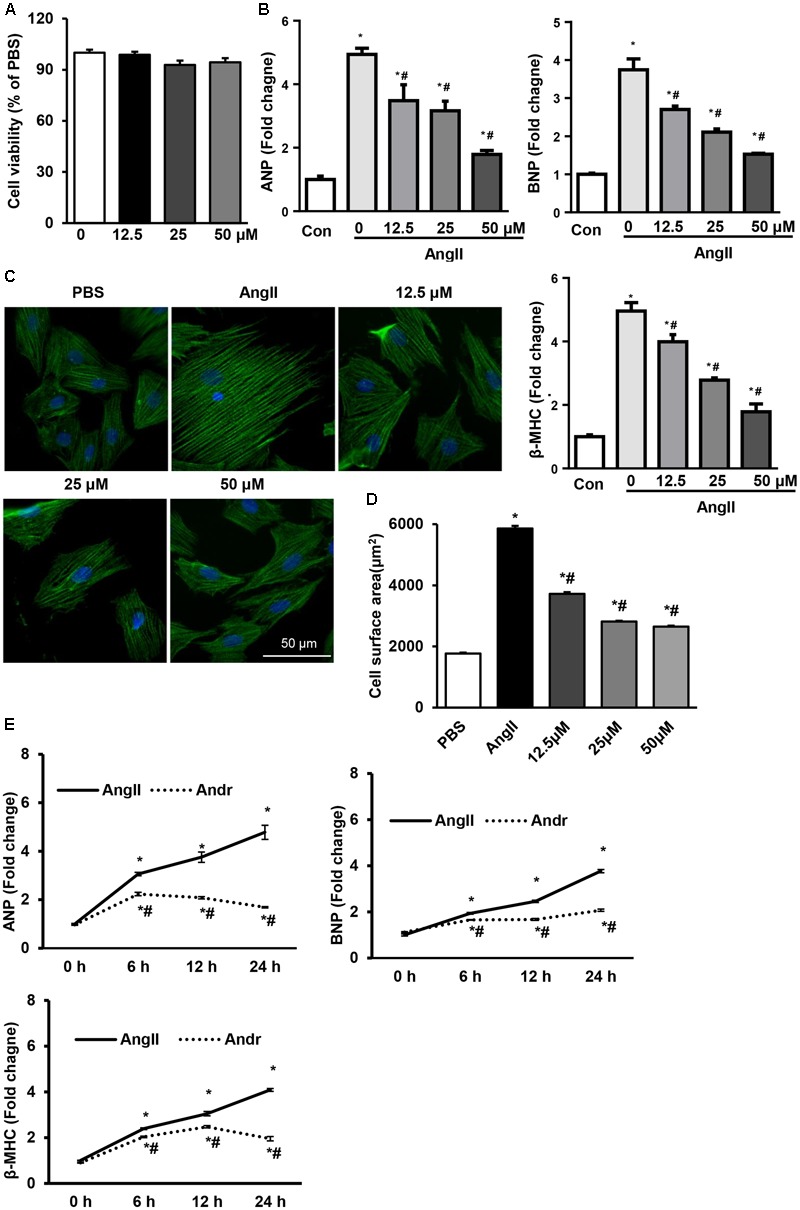
Andr suppresses Ang II-induced cardiomyocyte hypertrophy. Cardiomyocytes were stimulated with Ang II (1 μM) and treated with different concentrations of Andr (0, 12.5, 25, or 50 μM). **(A)** Cell viability was accessed by the cell counting kit-8 assay (*n* = 5). **(B)** The mRNA levels of ANP, BNP, and β-MHC in cardiomyocytes in the indicated groups (*n* = 6). **(C,D)** Immunofluorescence staining of α-actinin and the cell surface area of cardiomyocytes in the indicated groups (*n* = 5 samples, and *n* = 100+ cells per group). ^∗^*P* < 0.05 compared with the control group. #*P* < 0.05 vs. the Ang II group. **(E)** Cardiomyocytes were stimulated with Ang II (1 μM) and treated with Andr (50 μM) for 0, 6, 12, and 24 h. The mRNA levels of ANP, BNP, and β-MHC in cardiomyocytes in the indicated groups (*n* = 6). The results are presented as a fold change, and the results are normalized to GAPDH gene expression. ^∗^*P* < 0.05 compared with the corresponding 0 h group. ^#^*P* < 0.05 vs. the corresponding Ang II group.

To further determine whether Andr could attenuate cardiomyocyte hypertrophy, we treated cardiomyocytes with different concentrations of Andr (0, 12.5, 25, or 50 μM) and stimulated them with Ang II for 24 h. The cell counting kit assay-8 assay revealed that Andr (12.5, 25, or 50 μM) treatment did not affect cardiomyocyte viability (**Figure 4A**). Compared with the Ang II group, Andr treatment dramatically blunted the prohypertrophic effect of Ang II in a dose-dependent manner as indicated by the cell surface area, and expression of fetal genes (**Figures 4B–D**). Thus, 50 μM Andr was used in subsequent experiments.. After stimulating with Ang II for 6, 12, and 24 h, the cardiomyocytes exhibited gradually increased fetal gene expression, but Andr treatment nearly abolished this increase in the hypertrophic response (**Figure 4E**). These results indicate an important anti-hypertrophic role for Andr in cardiomyocytes.

### Andr Blocks MAPKs Signaling *in Vivo* and *in Vitro*

**FIGURE 5:**
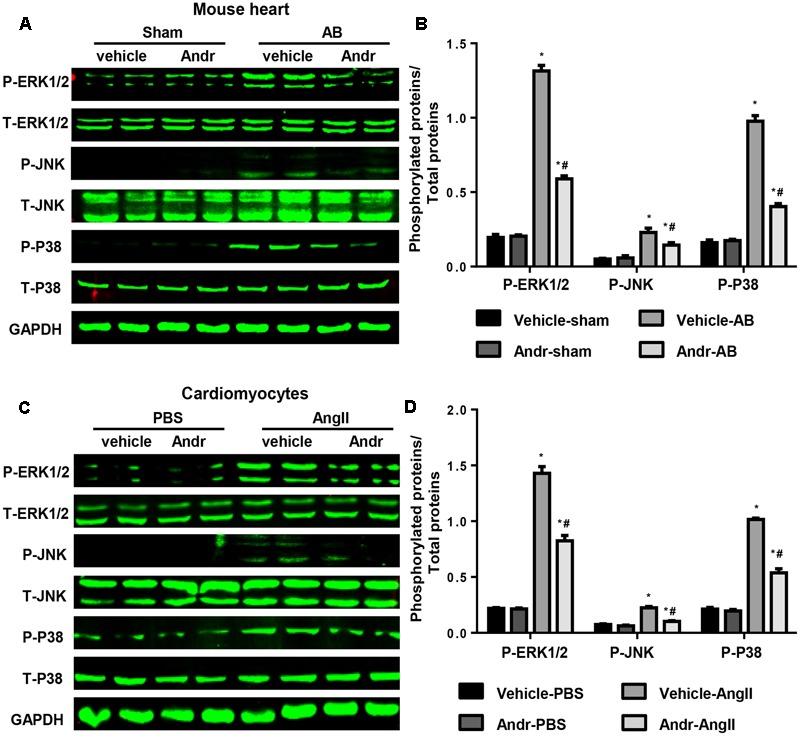
Andr blocks MAPKs signaling *in vivo* and *in vitro*. **(A)** Representative blots of phosphorylated (P-) and total (T-) ERK1/2, JNK, and P38 in the heart tissues of mice in the indicated groups (*n* = 6). **(B)** Comparison of expression among the indicated groups. ^∗^*P* < 0.05 compared with the corresponding sham group. #*P* < 0.05 vs. the veh-AB group. **(C)** Representative blots of phosphorylated (P-) and total (T-) ERK1/2, JNK, and P38 in the cardiomyocytes in the indicated groups (*n* = 6). **(D)** Comparison of expression among the indicated groups. ^∗^*P* < 0.05 compared with the corresponding PBS group. ^#^*P* < 0.05 vs. the vehicle-Ang II group.

We further gained insight into the molecular events mediating the anti-hypertrophic effect of Andr. Andr has been reported to exert its anti-inflammatory (Ren et al., 2016), anti-oxidant (Chen et al., 2014), antihyperglycemic (Li et al., 2015), and hepatoprotective properties (Pan et al., 2017) by regulating MAPKs signaling. Thus, we detected the protein expression of MAKPs. We found that MAPKs activation, including ERK1/2, JNK, and P38, was induced by pressure overload, but was strongly down-regulated in Andr-treated mice after 8 weeks of AB (**Figures 5A,B**). In line with this *in vivo* data, treatment cardiomyocytes with Andr resulted in dephosphorylation of ERK1/2, JNK, and P38 after Ang II stimulation (**Figures 5C,D**). The results imply that MAPKs may mediate the anti-hypertrophic effects of Andr.

### Andr-Mediated Cardioprotection Depends on the Inhibition of MAPKs in Cardiomyocytes

**FIGURE 6:**
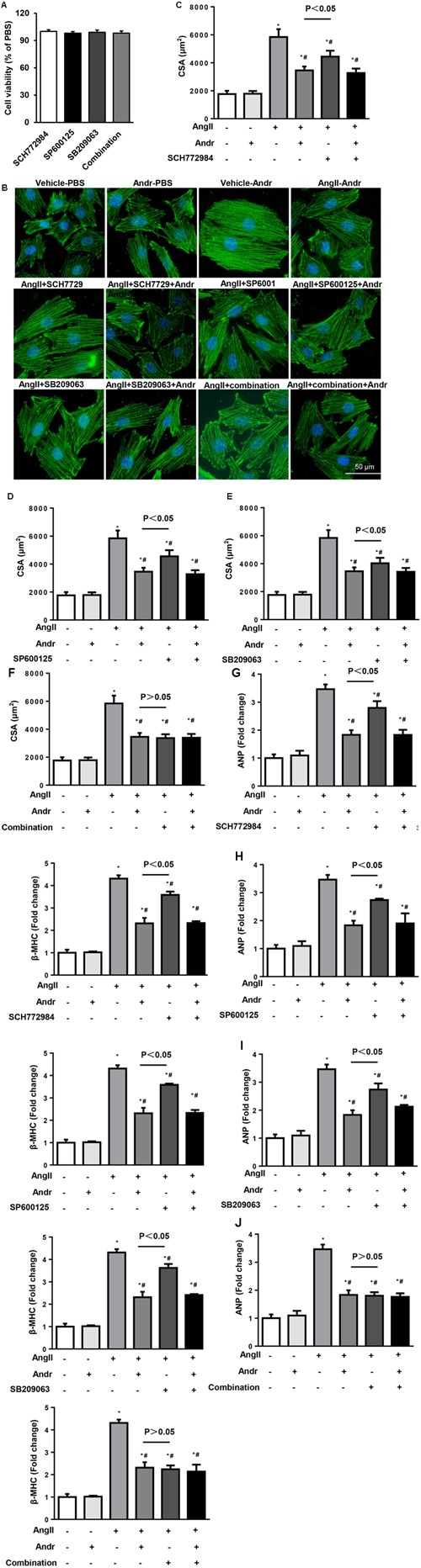
Andr-mediated cardioprotection depends on the inhibition of MAPKs in cardiomyocytes. Cardiomyocytes were treated with ERK1/2 inhibitor (SCH7729, 5 μM), JNK inhibitor (SP600125, 10 μM), and/or P38 inhibitor (SB209063, 10 μM) as well as stimulated with Ang II (1 μM) and treated with Andr (50 μM). **(A)** Cell viability was accessed by the cell counting kit-8 assay (*n* = 5). **(B–E)** Immunofluorescence staining of α-actinin and the cell surface area of cardiomyocytes in the indicated groups (*n* = 5 samples and *n* = 100+ cells per group). **(F–J)** The mRNA levels of ANP and β-MHC in cardiomyocytes in the indicated groups (*n* = 6). The results are presented as a fold change, and the results are normalized to GAPDH gene expression. ^∗^*P* < 0.05 compared with the control group. ^#^*P* < 0.05 vs. the Ang II group.

The effect of Andr on MAPKs was further confirmed by MAPKs inhibitors. Cardiomyocytes were treated with ERK1/2 inhibitor (SCH772984, 5 μM, Selleck), JNK inhibitor (SP600125, 10 μM, Sigma), and/or P38 inhibitor (SB209063, 10 μM, Medchem Express) as well as stimulated with Ang II. These inhibitors did not affect cardiomyocyte cell viability as shown in **Figure 6A**. None of these inhibitors could elicit a anti-hypertrophic response that was comparable to Andr when applied alone, but treatment with a combination of these three inhibitors did achieve a similar anti-hypertrophic response. Andr treatment could further augment the anti-hypertrophic effect of each inhibitor, as shown by the augmented reduction in surface area and fetal gene expression (**Figures 6B–J**). These findings suggest that suppression of all members of MAPKs (ERK1/2, JNK, and P38), underlies the anti-hypertrophic effects of Andr on cardiomyocytes.

### Andr Reduces Cardiac Fibroblast Activation via MAPKs

**FIGURE 7:**
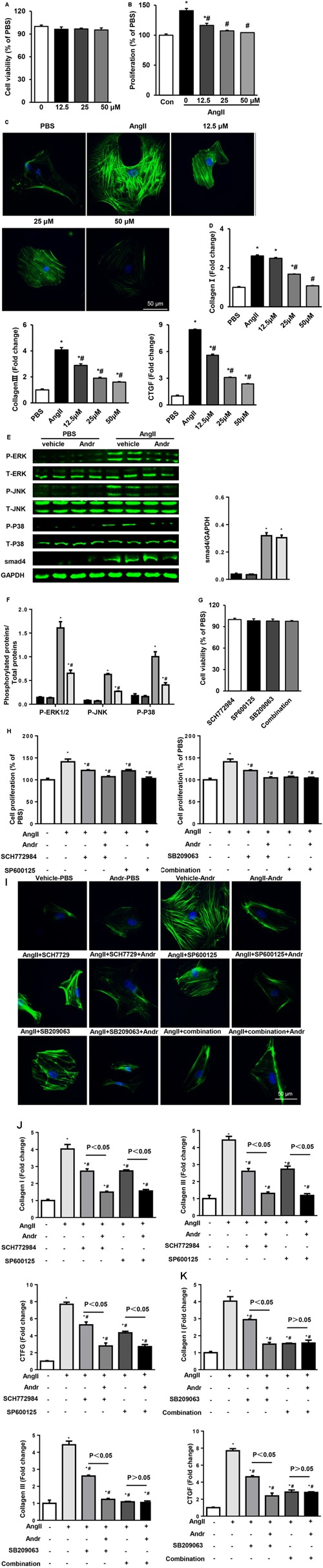
Andr reduces cardiac fibroblast activation via MAPKs. **(A–F)** Cardiac fibroblasts were treated with different concentrations of Andr (0, 12.5, 25, or 50 μM) and/or stimulated with Ang II (1 μM). **(A)** Cell viability was accessed by the cell counting kit-8 assay (*n* = 5). **(B)** Cell proliferation was accessed by the cell counting kit-8 assay (*n* = 5). **(C)** Immunofluorescence staining of α-SMA in the indicated groups. **(D)** The mRNA levels of collagen I, collagen III, and CTGF in cardiac fibroblasts in the indicated groups (*n* = 6). **(E)** Representative blots of phosphorylated (P-) and total (T-) ERK1/2, JNK, P38, and smad4 in the cardiac fibroblasts in the indicated groups (*n* = 6). **(F)** Comparison of expression among the indicated groups. ^∗^*P* < 0.05 compared with the corresponding PBS group. #*P* < 0.05 vs. the vehicle-Ang II group. **(G–J)** Cardiac fibroblasts were treated with ERK1/2 inhibitor (SCH7729, 5 μM), JNK inhibitor (SP600125, 10 μM), and/or P38 inhibitor (SB209063, 10 μM) as well as stimulated with Ang II (1 μM) and treated with Andr (50 μM). **(G)** Cell viability was accessed by the cell counting kit-8 assay (*n* = 5). **(H)** Cell proliferation was accessed by the cell counting kit-8 assay (*n* = 5). **(I)** Immunofluorescence staining of α-SMA in the indicated groups. **(J,K)** The mRNA levels of collagen I, collagen III, and CTGF in cardiac fibroblasts in the indicated groups (*n* = 6). The results are presented as a fold change, and the results are normalized to GAPDH gene expression. ^∗^*P* < 0.05 compared with the control group. ^#^*P* < 0.05 vs. the Ang II group.

Andr treatment ameliorated cardiac fibrosis at 8 weeks after AB in mice (shown in **Figure 3**). As cardiac fibroblasts play a major role in cardiac fibrosis, we then investigated whether Andr affects fibroblast activation. Cell counting kit-8 assay revealed that Andr (12.5, 25, or 50 μM) treatment did not affect fibroblast viability (**Figure 7A**). Ang II-induced fibroblast activation, proliferation, and function were inhibited by Andr treatment in a dose-dependent manner (**Figures 7A–D**). Andr also inhibited Ang II-induced MAPKs activation (including ERK1/2, JNK, and P38) in fibroblasts (**Figures 7E,F**), which was confirmed by ERK1/2 inhibitor, JNK inhibitor, and P38 inhibitor treatment (**Figures 7H–K**). However, Andr did not affect Ang II-induced smad4 expression (**Figures 7E,F**). Fibroblast viability was neither affected by single MAPKs inhibitor treatment nor treatment with a combination of MAPKs inhibitors (**Figure 7G**). These data suggest that the suppression of MAPKs in cardiac fibroblasts contributes to the anti-fibrosis effect of Andr on fibroblasts.

## Discussion

Pressure overload induces cardiomyocyte hypertrophy and fibroblast activation in cardiac tissue, resulting in cardiac hypertrophy and fibrosis followed by cardiac dysfunction in both human failure hearts (Gjesdal et al., 2011) and mouse models (Wu et al., 2015). Andr pharmacologically attenuated cardiac hypertrophy and fibrosis *in vivo*. In addition, our study also demonstrated that Andr ameliorated the Ang II-induced hypertrophic response in myocytes *in vitro*, as well as alleviated Ang II-induced fibroblast activation, proliferation, and function. Regarding the mechanism, we found that Andr suppressed the activation of ERK1/2, JNK, and P38 in both cardiomyocytes and fibroblasts ameliorating cardiac hypertrophy and fibrosis in the heart and improving cardiac function.

The mechanisms underlying the cardio-protective effects of Andr are not clear. In human patients with heart failure, MAPK signaling proteins have been reported to be hyperactive (Haque and Wang, 2017). Under physiological conditions MAPKs regulate cell proliferation and differentiation, whereas under pathological conditions, activated MAPKs induce the hypertrophic gene transcription (Liu and Molkentin, 2016). The downstream targets of MAPKs kinases include P38 kinase, JNK, and ERK1/2. Studies have shown that constitutive activation of ERK1/2 kinase contributes to concentric hypertrophy in cardiomyocytes (Kehat and Molkentin, 2010); ERK1/2 act upon nuclear factor of activated T-cells to mediate cardiac hypertrophy (Molkentin, 2013). JNK activation results in increased mitochondrion-associated apoptosis and fibrosis in the heart (Rose et al., 2010). Activation of JNK contributes to restrictive cardiomyopathy and promotes fibrosis in the heart (Petrich et al., 2004). P38 is also involved in myocyte growth. P38 inhibitors (SB203580 or SB202190) suppressed hypertrophic stimuli induced myocyte growth and dominant negative P38 delivered by adenoviruses (Zechner et al., 1997; Liang and Molkentin, 2003). Our previous studies have reported that many molecular and plant extracts ameliorate pressure overload-induced cardiac hypertrophy via MAPKs inhibition (Wu et al., 2017b). ATF3 exerts negative feedback on the ERK and JNK pathways to modulate cardiac remodeling (Zhou et al., 2011). Mnk1 prevents cardiac hypertrophy by inhibiting the Ras/ERK pathway (Yuan et al., 2016). Other plant-derived inhibitors, such as geniposide, indole-3-carbinol, and baicalein, also target on MAPKs signaling proteins (Wu et al., 2017b). These findings do not indicate whether a particular molecule is definitively ‘bad’ or ‘good.’ By focusing on network interactions rather than signal molecules, we may have a better chance at influencing the outcome. Previous reports have indicated that Andr exerts various effects by regulating MAPKs. Andr ameliorates rheumatoid arthritis by inhibiting MAPKs pathways (Li Z.Z. et al., 2017). Andr protects against ischemic stroke in rats by regulating the MAPKs signaling cascade (Yen et al., 2016). Andr reduces IL-2 production in T-cells by interfering with NFAT and ERK activation (Burgos et al., 2005; Carretta et al., 2009). Conversely, Andr induces Nrf2 and HO-1 in astrocytes by activating p38 and ERK (Wong et al., 2016). Andr inhibits the growth of human T-cell acute lymphoblastic leukemia Jurkat cells by upregulating of P38 pathways (Yang et al., 2016). These results indicate that Andr differentially regulates MAPKs in different cell types. Our results revealed that Andr inhibits the three terminal effectors of the MAPKs signaling cascade following induction by hypertrophic stimuli in both cardiomyocytes and fibroblasts. Using ERK1/2, JNK, and P38 inhibitors, we further demonstrated that Andr exerts cardio-protective effects by inhibiting both ERK, JNK, and P38. Andr exhibits similar inhibition efficiency for ERK, JNK, and P38. As our result in **Figure 6** shows, single MAPK protein inhibition exerts similar anti-hypertrophic effect. Andr equally augmented these anti-hypertrophic responses. Direct evidence of the inhibition efficiency of Andr for ERK, JNK, and P38 requires further study.

Cardiac fibrosis is a major feature of hypertrophic cardiomyopathy and contributes to ventricular dysfunction and life-threatening arrhythmia (Travers et al., 2016). Our *in vivo* study showed that Andr treatment abated the pressure overload-induced fibrotic response. Cardiac fibroblasts contribute to the heart’s response to various forms of injury. After myocardial injury, the expression of various pro-fibrotic factors is up-regulated in fibroblasts, leading to increased fibroblast cell proliferation and ultimately, its transition to the myofibroblast phenotype (Kurose and Mangmool, 2016). Under these conditions, a subset of activated myofibroblasts acquire new phenotypic characteristics, including expression of the contractile protein α-SMA, and contribute to pathological cardiac remodeling (Tomasek et al., 2002). Considering the key role of fibroblasts in cardiac fibrosis, we wondered whether Andr could directly target fibroblasts. Our *in vitro* results showed that Andr ameliorates Ang II-induced fibroblast activation, proliferation, and function I. The AT1R mediates many effects of Ang II in fibroblasts, including cell proliferation, cell migration, and the induction of extracellular matrix protein synthesis (Gao et al., 2009). AT1R activation results in the G-protein-dependent activation of MAPKs, which leads to the activation and expression of collagen in fibroblasts (Dostal et al., 2015). Ang II is also involved in TGFβ/smad signaling. Activation of AT1R by Ang II induces the expression TGF-β1 (Ma et al., 2012). We found that Andr suppressed the activation of MAPKs. MAPK inhibition by a combination of specific inhibitors exerted the same protective effects as Andr. These results indicate that the anti-fibrotic effect of Andr depends the inhibition of MAPKs. In contrast, Andr did not affect smad4 expression, indicating that smad signaling may not be a target of Andr.

In the *in vivo* study, 25 mg/kg/day Andr was used from 1 week after surgery to 8 weeks after surgery. A pharmacokinetic study reported that the blood concentration–time curve of Andr by oral gavage in rats (10 mg/kg) was fitted to the one-compartment model, in which the blood concentration of Andr increases sharply to 1.6 μg/mL in 100 min and gradually decreases within 600 min (Suo et al., 2007). The 25 mg/kg/day oral dose via gavage that was used in our study could maintain the blood Andr concentration at a certain level. However, accurate pharmacokinetic measurement in mice requires further study.

## Conclusion

We documented the effective inhibition of the MAPKs signaling pathway by Andr treatment in both cardiomyocytes and fibroblasts and showed that MAPKs mediates the anti-hypertrophic effect of Andrin heart tissue. Cardiac hypertrophy due to stress, such as pressure overload, often culminates in heart failure and is associated with adverse cardiovascular events. Classical pharmacological treatment strategies for heart failure are ineffective in a number of patients. Although the effect of Andr on human cardiac hypertrophy and heart failure has not yet been reported, these observations in mice are critical for the development of treatment strategies for cardiac hypertrophy and heart failure.

## Author Contributions

QW, QT, and WD contributed to the conception, and design of the experiments; QW, JN, and WD carried out the experiments; NZ and HL analyzed the experimental results, and revised the manuscript. QW and WD wrote and revised the manuscript.

## Conflict of Interest Statement

The authors declare that the research was conducted in the absence of any commercial or financial relationships that could be construed as a potential conflict of interest.

## Abbreviations

Andr: andrographolide
ANP: atrial natriuretic peptide
AT1R: Ang II type 1 receptor
BNP: brain natriuretic peptide
CTGF: connective tissue growth factor
dP/dt max: maximal rate of pressure development
dP/dt min: minimal rate of pressure decay
ERK: extracellular signal-regulated kinase
GAPDH: glyceraldehyde-3-phosphate dehydrogenase
H&E: hematoxylin and eosin
HO-1: heme oxygenase 1
HR: heart rate
LV: left ventricle
LVEDd: LV end-diastolic diameter
LVESd: LV end-systolic diameter
LVPWd: left ventricular posterior wall thickness
MAPK: mitogen-activated protein kinase signaling
MHC: myosin heavy chain
PSR: picrosirius red
TGFβ: transforming growth factor β
α-SMA: α-smooth muscle actin
